# Feasibility of Digital Memory Assessments in an Unsupervised and Remote Study Setting

**DOI:** 10.3389/fdgth.2022.892997

**Published:** 2022-05-26

**Authors:** David Berron, Gabriel Ziegler, Paula Vieweg, Ornella Billette, Jeremie Güsten, Xenia Grande, Michael T. Heneka, Anja Schneider, Stefan Teipel, Frank Jessen, Michael Wagner, Emrah Düzel

**Affiliations:** ^1^German Center for Neurodegenerative Diseases (DZNE), Magdeburg, Germany; ^2^Clinical Memory Research Unit, Department of Clinical Sciences Malmö, Lund University, Lund, Sweden; ^3^neotiv GmbH, Magdeburg, Germany; ^4^Institute of Cognitive Neurology and Dementia Research (IKND), Otto-Von-Guericke University, Magdeburg, Germany; ^5^German Center for Neurodegenerative Diseases (DZNE), Bonn, Germany; ^6^Department of Neurodegeneration and Geriatric Psychiatry, University of Bonn, Bonn, Germany; ^7^Department of Psychosomatic Medicine, Rostock University Medical Center, Rostock, Germany; ^8^German Center for Neurodegenerative Diseases, Rostock, Germany; ^9^Department of Psychiatry, University Hospital Cologne, Cologne, Germany; ^10^Institute of Cognitive Neuroscience, University College London, London, United Kingdom

**Keywords:** digital cognitive assessment, remote and unsupervised cognitive assessment, episodic memory, participant retention, smartphone-based cognitive assessments

## Abstract

Sensitive and frequent digital remote memory assessments via mobile devices hold the promise to facilitate the detection of cognitive impairment and decline. However, in order to be successful at scale, cognitive tests need to be applicable in unsupervised settings and confounding factors need to be understood. This study explored the feasibility of completely unsupervised digital cognitive assessments using three novel memory tasks in a Citizen Science project across Germany. To that end, the study aimed to identify factors associated with stronger participant retention, to examine test-retest reliability and the extent of practice effects, as well as to investigate the influence of uncontrolled settings such as time of day, delay between sessions or screen size on memory performance. A total of 1,407 adults (aged 18–89) participated in the study for up to 12 weeks, completing weekly memory tasks in addition to short questionnaires regarding sleep duration, subjective cognitive complaints as well as cold symptoms. Participation across memory tasks was pseudorandomized such that individuals were assigned to one of three memory paradigms resulting in three otherwise identical sub-studies. One hundred thirty-eight participants contributed to two of the three paradigms. Critically, for each memory task 12 independent parallel test sets were used to minimize effects of repeated testing. First, we observed a mean participant retention time of 44 days, or 4 active test sessions, and 77.5% compliance to the study protocol in an unsupervised setting with no contact between participants and study personnel, payment or feedback. We identified subject-level factors that contributed to higher retention times. Second, we found minor practice effects associated with repeated cognitive testing, and reveal evidence for acceptable-to-good retest reliability of mobile testing. Third, we show that memory performance assessed through repeated digital assessments was strongly associated with age in all paradigms, and individuals with subjectively reported cognitive decline presented lower mnemonic discrimination accuracy compared to non-complaining participants. Finally, we identified design-related factors that need to be incorporated in future studies such as the time delay between test sessions. Our results demonstrate the feasibility of fully unsupervised digital remote memory assessments and identify critical factors to account for in future studies.

## Introduction

Sensitive and frequent remote cognitive assessments via mobile devices hold the promise to facilitate the detection of cognitive impairment and decline, where snapshots in time at irregular or symptomatic visits to a doctor fail to provide a full picture of the cognitive state of a person. Established cognitive assessment batteries require in-clinic assessments with trained clinical neuropsychologists and time-limited testing sessions, which complicate the examination of long-term memory function across the time course of several hours or even days. Furthermore, repeated memory testing is often limited by the number of available parallel test versions, and commonly results in considerable practice effects (1). Smartphone-based remote cognitive assessments hold the potential to facilitate large-scale high-frequency assessments, and to test cognitive function over the span of days, weeks and even years (2, 3). However, for remote cognitive assessments to be effective at scale, they need to work in a remote and unsupervised setting. One major difference between in-clinic and remote assessments is the lack of a standardized testing environment, and many factors have the potential to hamper measures of cognition (4). For example, different hardware, testing time or environment and their respective distractions, as well as individual circumstances like illness or sleep deprivation may influence cognitive performance. Moreover, one of the biggest hurdles for remote digital cognitive testing is participant retention, as recent work has highlighted high rates of dropout in remote digital health studies (5).

Here, we relied on three recently established memory paradigms, that have all been translated into the neotiv-App (neotiv.com), which allows remote testing via smartphones or tablet computers. First, the Mnemonic Discrimination Task for Objects and Scenes (MDT-OS) is a short-term memory task designed to assess pattern separation (6). Second, the Object-In-Room Recall (ORR) task was developed to gauge pattern completion by short- and long-term object-scene associations [for a discussion of the principles see (7)]. Third, the Complex Scene Recognition task (CSR) (8) is a long-term photographic scene recognition memory test. All three paradigms have been shown to rely on critical brain networks for human episodic memory in recent functional magnetic resonance imaging studies, and two of them have been associated with imaging and fluid biomarkers of AD pathology (9–11).

In the present study, digital remote memory assessments are performed by a large citizen science cohort over a maximum period of 12 weeks. We evaluate psychometric properties of these assessments such as test-retest reliability and external validity regarding age effects and subjective memory impairment (12, 13). Using fully independent parallel test versions for each memory task, we aim to reduce practice effects due to weekly repetitions across 12 weeks. Finally, using survival analyses we identify factors that are associated with long-term participation in our study including active participation in memory tasks.

## Materials and Methods

### Recruitment and Procedure

The study was advertised via the online Citizen Science platform *BürgerSchaffenWissen* (www.buergerschaffenwissen.de) which is funded by the German Ministry of Research and Education. In addition, the study was advertised via a specific project website (exploring-memory.org) as well as local advertisements. Via these channels, participants were invited to register for the study and download the neotiv-App from the respective app stores onto their personal smartphone or tablet computer (iOS or Android devices). When starting the app, participants gave written consent. The study was approved by the ethics committee of the Otto-von-Guericke-University Magdeburg.

In the following 12 weeks, participants were asked to complete weekly memory assessments where each participant was randomly assigned to one of the three memory tasks. Each of the assessments consisted of a 2-phase session separated by at least 24 h. The two phases were either two halves of mnemonic discrimination, or encoding and retrieval phases of complex scene recognition and object-in-room recall (see details of the tasks below). Every phase took <10 min. Thus, overall participants could complete up to 12 sessions within a period of ~3 months. In order to minimize potential practice effects from repeated testing, 12 independent parallel test versions for each memory task were used. After each task completion, participants indicated their subjective task performance and their concentration level on a 5-point scale (1 = very bad, 2 = bad, 3 = middling, 4 = good and 5 = very good), and whether they had been distracted throughout the task (yes/no).

Push notifications were used to notify individuals about available tasks and to remind them daily for five consecutive days in case the tasks had not been initiated. At the beginning of each test session, participants were asked to go to a quiet environment, wear their glasses if needed and to adjust their screen's brightness to see the pictures clearly. They also received a short practice session for the initial test session as well as all future sessions.

When starting the very first session, individuals were shown several images comparable to those used in the memory tasks to get an estimate of their perceptual discrimination performance. Instead of a delayed presentation, individuals had to indicate whether there were differences between picture pairs when shown side by side.

**Figure 1:**
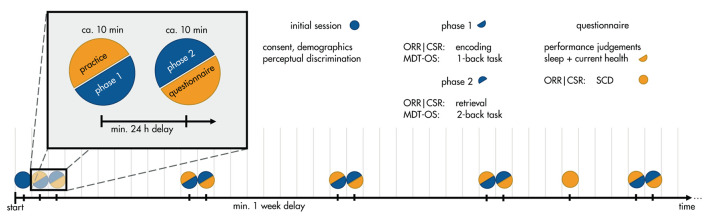
Timeline of the study protocol. Participants enlisted for a 12-week study of weekly memory assessments. In a between-subjects design, participants were assigned to one of three tasks: Mnemonic Discrimination Test for Objects and Scenes (MDT-OS), Objects-in-Room-Recall (ORR), or Complex Scene Recognition (CSR). In the initial session, they gave consent, demographic information, and did a brief perceptual discrimination task. Each week, they received a short training session, followed by phase 1 of their respective task: encoding for ORR and CSR, and 1-back task for MDT-OS. 24 h after finishing phase 1, they were notified that the next phase was available, and could perform it straightaway or postpone if inconvenient; that is, there was a minimum delay of 24 h, but it was often extended by the participants (see Important Factors for Unsupervised Assessments). Phase 2 consisted of retrieval for ORR and CSR, and 2-back task for MDT-OS. It was followed by judgements regarding concentration and distraction throughout the task, current health (cold symptoms) and sleep quality. In week 4, participants of ORR and CSR received an additional questionnaire about subjective cognitive complaints (SCD).

In addition, participants were asked to complete weekly short questionnaires regarding sleep (average duration, bedtime, time to fall asleep, wake-up time), and cold symptoms (flu vaccination, respiratory illness, fever, how affected by symptoms). Finally, two of the study groups were asked to voluntarily complete a more detailed questionnaire on subjective cognitive complaints in the 4^th^ week of the study (14). Their judgment of whether their memory capacity has become worse during the 5 years preceding the study was used as a grouping variable for subjective memory decline. The full study timeline can be seen in Figure 1.

### Memory Tasks

#### Mnemonic Discrimination of Objects and Scenes (MDT-OS)

**Figure 2:**
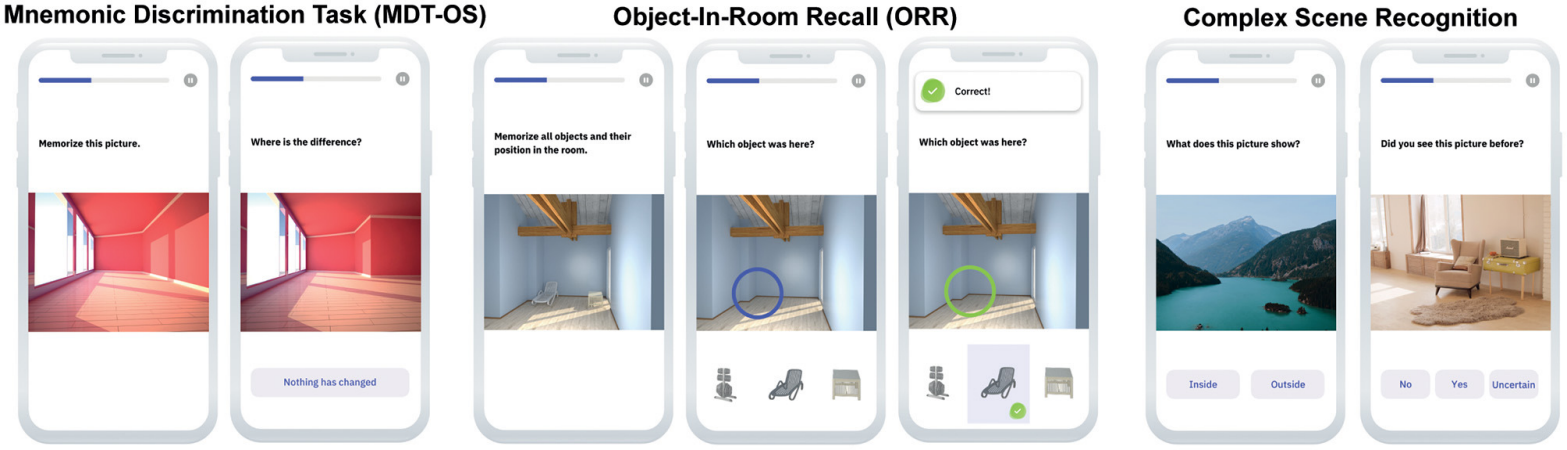
Digital memory assessments. Mnemonic Discrimination Task for Objects and Scenes (MDT-OS), Objects-in-Room Recall (ORR) Test and Complex Scene Recognition Test (CSR).

In this continuous recognition task, individuals were presented with 3D rendered computer-generated images of various indoor objects and empty rooms, which were either exactly repeated, or slightly altered (see Figure 2). Participants had to indicate whether an image was an identical repetition (tap on a button), or had been modified (tap on the location of change). One session consisted of 64 image pairs (32 object pairs, 32 scene pairs), half of which were modified, and half of which were repeated. In order to reduce participant burden, one session was split into two phases and completed on two consecutive days following a 24-h delay. The first phase was presented as a one-back task while the second phase was presented as a two-back task. The MDT-OS has been designed to tax hippocampal pattern separation; a memory mechanism needed to discriminate between similar memories. Earlier studies using functional magnetic resonance imaging have shown that especially subregions in the human medial temporal lobe are involved in this task (6, 9, 11).

#### Object-in-Room Recall (ORR)

In this task, participants had to memorize a spatial arrangement of two objects within a room. Following the encoding phase, a blue circle highlighted the position of one of the objects in an empty room and the participant had to identify the correct object from a selection of three in an immediate retrieval phase (see Figure 2). Of the three possible objects, one was the correct object at this very position (target), one was the object from the same room but the wrong position (correct source distractor), while the third had been shown in another room before (incorrect source distractor). They learned 25 such object-scene associations. After 24 h, the participant was notified via push notification to complete an identical but delayed retrieval phase with a randomized stimulus order. The ORR has been designed to tax hippocampal pattern completion, a memory mechanism needed to restore full memories from partial cues (7). In the test, the assessment of recall is graded and allows to separate correct episodic recall from incorrect source memory. Thus, correct recall excludes the choice of an object that was present in the same room but at a different location (wrong source memory for specific location) and an object that was not present in the room but nevertheless associated with the objects belonging to the room during encoding (wrong source memory for overall location).

#### Complex Scene Recognition (CSR)

In this task, participants had to memorize 60 photographic indoor and outdoor scenes while classifying them into each category. After 24 h participants were notified via push notification to complete the retrieval phase. Here, they were shown the same pictures again intermixed with 30 novel pictures, and needed to make old/new/uncertain recognition memory decisions (see Figure 2). This task has been designed to tax a more widespread episodic memory network for encoding and retrieval as has been shown in earlier fMRI studies using the same task (8, 10).

### Initial Filtering Procedures

The study began in January 2019 and for this manuscript, we analyzed data up until the data release on 17th January 2020. First of all, because memory tasks were presented in two phases, we only included participants that had completed at least one full session, i.e., both phases. Age, screen size, time of day, time since baseline, and time between encoding and retrieval were prepared as predictors for subsequent modeling. Of the individual trials of each memory task, the following outcome measures were calculated. For the MDT-OS, we calculated the corrected hit rate to correct for response bias by subtracting the false alarm rate (percentage of repeated images that were identified as novel) from the hit rate (the percentage of repeated images that were identified as repeated). For the ORR, we calculated the 24-h delayed retrieval accuracy. For the CSR, again the corrected hit rate was calculated by subtracting the false alarm rate (percentage of wrongly remembered images) from the hit rate (percentage of correctly remembered images). For linear-mixed effects models, only subjects with more than two available sessions were included and only sessions with responses to at least 80% of trials within a session.

### Statistical Analyses

We used linear-mixed effects models (lme4 package in R, version v3.6.0, http://www.r-project.org) to analyze longitudinal effects of age, sex, time since baseline (i.e., practice effects), time of day, time to retrieval and screen size of the mobile device associated with the three memory tasks. *T*-tests were used to assess significance of all fixed-effects predictors (*p* < 0.05, unc.). In our stepwise approach, we first estimated random intercept models accounting for linear effects of all above-mentioned factors. Afterwards, quadratic effects for all predictors related to time were included to enable testing of potential non-linear effects. Next, all non-significant effects with *p* > 0.1 were excluded and interaction terms were included. From here, we excluded all non-significant effects and interactions, and assessed their superiority over the first models using the Akaike Information Criterion (AIC). If the first model was superior, it was also reduced to significant factors. Finally, using the factors of the superior models, we compared the random intercept models to random slope models using AIC. These superior models are reported as final models in the results section. In addition, we tested the effect of cold symptoms (individuals who reported a cold infection vs. those who did report no infection), the effect of average night sleep duration, and the effect of perceived subjective memory decline in MDT-OS and ORR (individuals who reported subjective memory decline throughout the 5 years preceding the study vs. those who did not report such perceived decline) in separate models. A full table containing all calculated models can be found in the Supplementary Material. Note, that in this feasibility study we did not focus on specific effects but explored the contributions of demographics, design and acquisition related factors as well as their consistency across tasks to support validity of unsupervised cognitive assessments.

Participant retention analysis (survival analysis) was conducted using the total number of completed sessions as the outcome metric. We did not use the longest time participants stayed in the study, but rather their interaction, i.e., the number of completed tasks. That is, picture someone who participated in week one, then did nothing for 10 weeks, but took another test in week 12 – their retention time would be 12 weeks, but their data contribution only two sessions. In contrast, someone who performed the first six sessions would have contributed three times as much, but their retention time would have been half as long. Log-rank tests were used to compare the differences in the number of completed sessions across memory paradigms, sex, age group and subjective task performance. For sex and memory paradigms, we used the respective categories (female/male and MDT-OS/ORR/CSR). For the retention analysis with respect to age, we grouped the data according to 33% and 66% quantiles, resulting in three groups with participants <50 years, between 50 and 60, and >60 years. For mean subjective task performance, we used cut-offs of participants' performance ratings resulting in 3 groups (low: ratings up to 2, mid: ratings between 2 and 3, high: ratings above 3; scale 1: very bad – 5: very good). We used a no-censoring approach where the last completed task was considered a participant leaving the study, and Kaplan-Meier plots to visualize a summary of the respective effects.

In order to determine the retest reliability of the different memory tasks, we calculated the Pearson correlation coefficient of outcomes of the two subsequent test sessions 2 and 3 to avoid effects of general accommodation to the study in session 1. Additionally, we calculated the Pearson correlation coefficient between the averages of two sessions (mean of sessions 1 and 2 vs. mean of sessions 3 and 4) to minimize day-to-day variability.

## Results

### Participants

**Figure 3:**
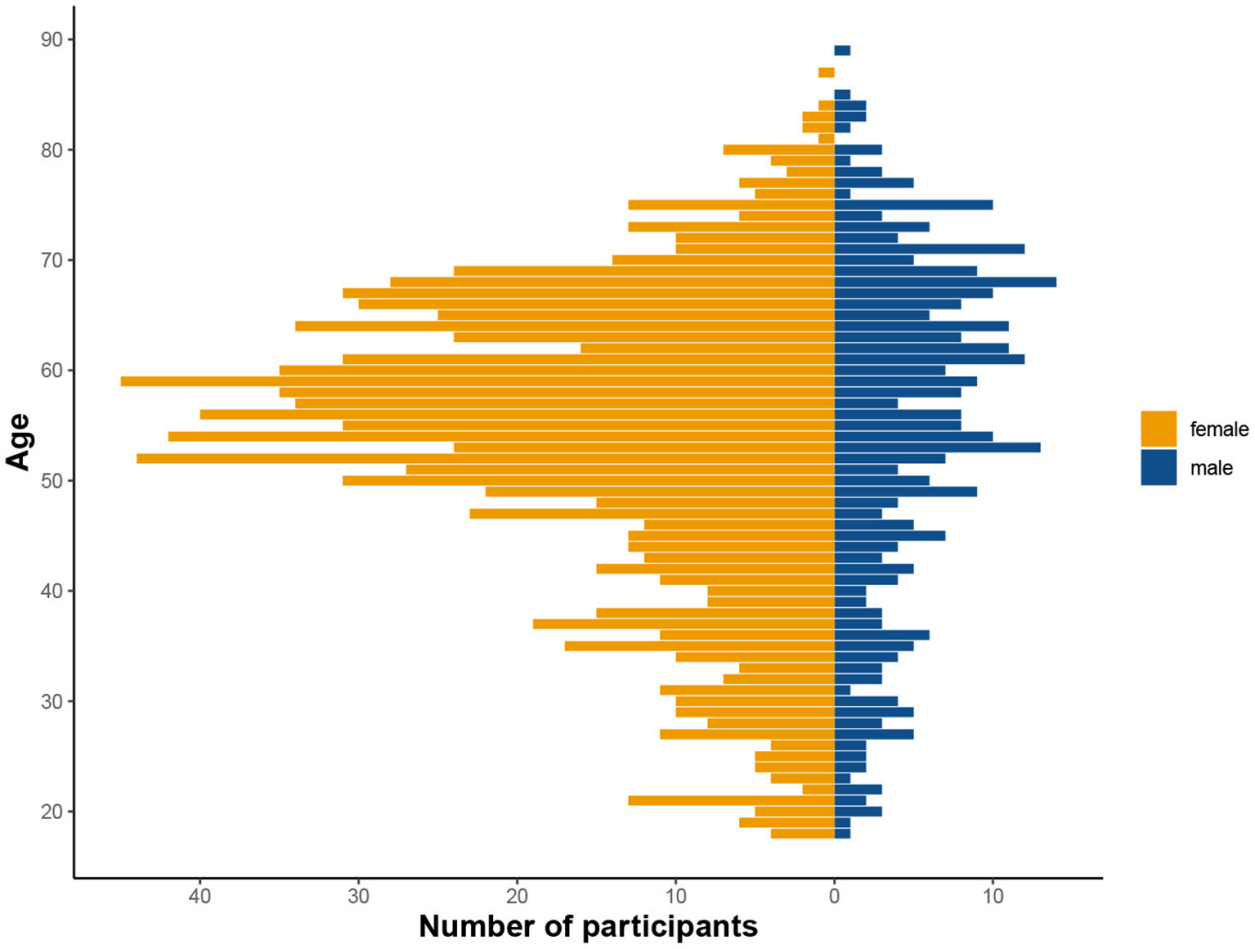
Distribution of age and sex in the tested cohort. Considerably more women than men participated in the study, as is not uncommon in citizen science projects (see Discussion).

Originally, there were 2,076 individuals that downloaded the app and registered for the study. Given the self-enrolling character of the study, many people will check out the app swiftly to decide about their participation. Therefore, we set a minimum of one completed session for inclusion into the study. Consequently, a total of 1,407 individuals participated in the study (mean age = 53.9, age range = 18–89 years, 75.6 % female, see Table 1 and Figure 3), and were allocated to three different sub-studies in a pseudorandomized fashion which only differed with respect to the memory task (MDT-OS/ORR/CSR). Following the completion of a study, participants were offered to participate in one more study using a different task. One hundred thirty-eight individuals decided to participate in another task after finishing; 77 took part in both MDT-OS and ORR, and 61 in both ORR and CSR. Thus, 447 individuals were assigned to the Mnemonic Discrimination task, 683 individuals to the Object-In-Room Recall task and 415 individuals to the Complex Scene Recognition task. Note that the combined total of the individual tasks is 1,545 due to the overlapping participants. Demographic details of each task group, and for the actual total of 1,407 individuals can be found in Table 1.

**Table 1:** Demographics, task-specific questionnaire data, and retention.

			**Mnemonic discrimination** **(*N* = 447)**	**Object-in-room recall** **(*N* = 683)**	**Complex scene recognition** **(*N* = 415)**	**Total** **(*N* = 1407)**
Age (years)	Mean (SD)		54.4 (14.0)	54.7 (13.7)	54.0 (14.8)	53.9 (14.3)
	Range		19–87	18–87	18–89	18–89
Sex (N)	Female		349 (78.1%)	489 (71.6%)	340 (81.9%)	1,064 (75.6%)
	Male		98 (21.9%)	194 (28.4%)	75 (18.1%)	343 (24.4%)
Subjective memory decline (N)	Stable		208 (46.5%)	333 (48.8%)	NA	500 (35.5%)
	Declining		67 (15.0%)	112 (16.4%)	NA	151 (10.7%)
	Missing		172 (38.5%)	238 (34.8%)	415 (100%)	756 (53.7%)
Perceptual discrimination	Mean (SD)		90.9% (11.7%)	89.9% (12.8%)	90.8% (11.9%)	90.5% (12.3%)
Screen diagonal (cm)	Mean (SD)		13.7 (3.99)	14.1 (4.43)	13.6 (4.08)	13.8 (4.18)
Delay between phases (hours)	Mean (SD)		41.7 (14.0)	40.2 (13.4)	38.8 (13.0)	40.2 (13.5)
Subjective task performance (1: very bad – 5: very good)	Mean (SD)	Phase 1 Phase 2	3.3 (0.6) 2.9 (0.6)	4.4 (0.5) 2.6 (0.7)	4.2 (0.5) 3.3 (0.6)	4.0 (0.7) 2.9 (0.7)
Concentration (1: very bad – 5: very good)	Mean (SD)	Phase 1 Phase 2	3.7 (0.5) 3.4 (0.6)	4.2 (0.6) 3.7 (0.7)	4.1 (0.5) 3.9 (0.6)	4.0 (0.6) 3.6 (0.7)
Distraction (In % of sessions)	Mean (SD)	Phase 1 Phase 2	27.8% (31.5%) 27.2% (35.9%)	23.7% (30.1%) 17.2% (30%)	21.1% (27.9%) 15.4% (26.7%)	24.7% (30.2%) 19.9% (31.6%)
Duration (Min)	Mean (SD)	Phase 1 Phase 2	9.82 (1.89) 10.9 (2.29)	11.4 (1.87) 4.41 (0.987)	9.64 (2.20) 6.08 (2.18)	10.4 (2.08) 6.74 (3.18)
Retention					
Time (days)	Mean (SD)		44.8 (34.3)	47.4 (34.6)	47.5 (34.4)	44.2 (33.6)
Number of complete sessions	Mean (SD)		3.81 (3.30)	4.74 (3.75)	4.70 (3.74)	4.23 (3.49)
Compliance with protocol	Mean (SD)		73.8% (24.3%)	78.6% (23.9%)	78.4% (22.3%)	77.5% (23.3%)

*Note, that the combined total of the individual tasks is 1,545 due to 138 participants who took part in two paradigms each. The presented total, here, refers to the actual number of participants (N = 1,407). Data on subjective cognitive complaints was not collected in individuals performing the CSR task. Context information (concentration, distraction, subjective performance) was collected for both phases (encoding and retrieval in ORR and CSR, and two halves of MDT-OS) and is presented separately. Retention expresses how much participants contributed to the task with a possible maximum of 12 completed weekly sessions. Compliance with protocol refers to the percentage of tests taken within the participants' total time in the study, i.e., full contribution is reached with the same number of sessions as weeks in the study*.

### Age Effects and Subjective Memory Decline

**Figure 4:**
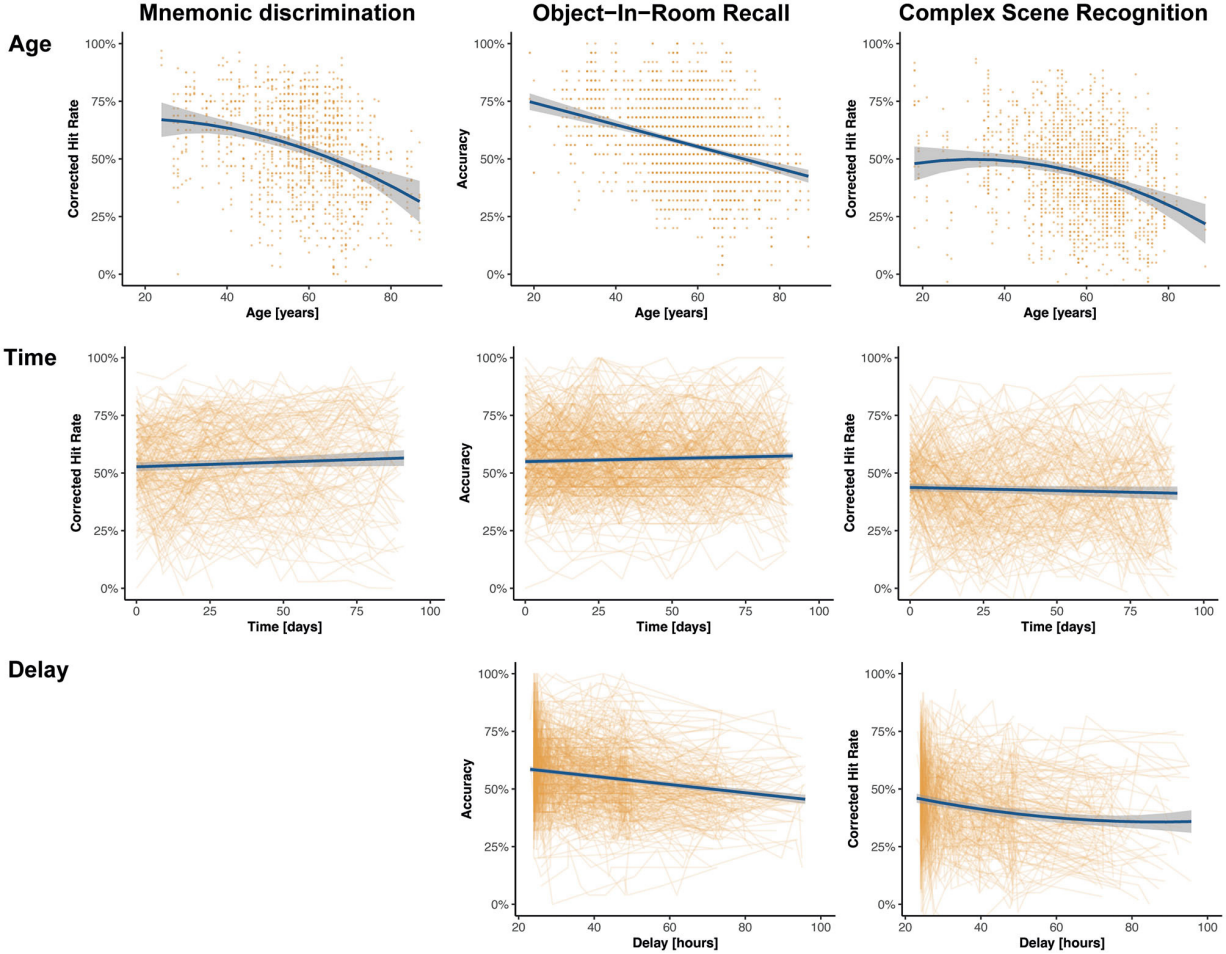
Effects of interest of linear-mixed effects models across memory paradigms. The model predictions are shown in blue, and the non-adjusted original data points are shown in orange. The top panel shows the effect of age on memory performance where higher age is associated with worse memory performance across all paradigms. The effect is linear in ORR, quadratic in CSR, and trending quadratic in MDT-OS. The middle panel shows the effect of time since baseline on memory performance across all tasks, suggesting minor linear practice effects for MDT-OS und ORR, and no practice effects for CSR. The bottom panel shows that with longer delays between encoding and retrieval, performance gets worse in ORR and CSR. Note that MDT-OS is a continuous recognition paradigm, i.e., encoding and retrieval are not separated across the two phases, thus, not surprisingly the delay does not affect performance.

Memory functioning deteriorates with age and as the participants of this study covered a wide range of the adult life span (18–89 years), we analyzed memory differences across individuals of different ages. Using linear-mixed regression, we found significant linear effects of age in ORR, and significant quadratic effects in CSR and trending quadratic in MDT-OS (see top panel of Figure 4), that is, performance declined with increasing age. We observed no sex differences. Furthermore, participants of two of the studies had been asked whether they subjectively worry that their memory is declining (in MDT-OS and ORR; see Table 1). Splitting individuals in groups of subjectively stable and declining individuals while excluding those that left the question unanswered, linear-mixed regression showed that individuals with subjective memory decline had slightly worse memory in MDT-OS. However, we did not observe a corresponding effect on memory decline over the 12 weeks of study duration. Similarly, we found no significant difference between subjectively stable and declining subjects in ORR. Likewise, an individual's usual night's length of sleep, or flu/cold symptoms did show no effect on memory performance in neither of the three memory paradigms (see Supplementary Material). All results are presented in Table 2.

**Table 2:** Significant results of the final linear mixed-effects models per memory task.

	**Mnemonic discrimination**					**Object-in-room recall**					**Complex scene recognition**				
**Predictors**	**Est**.	**Std. Beta**	**CI**	***p***	**Est**.	**Std. Beta**	**CI**	***p***	**Est**.	**Std. Beta**	**CI**	***p***
(Intercept)	0.587	0.038	0.348–0.826	<0.001	0.864	−0.041	0.807–0.920	<0.001	0.536	−0.015	0.366–0.707	<0.001
Time	0.0004	0.058	0.00005–0.0008	0.027	0.0003	0.047	0.0001–0.0004	0.002	−0.0003	−0.041	−0.0006–0.00004	0.083
Age	0.002	−0.389	−0.006–0.011	0.592	−0.005	−0.353	−0.006–0.004	<0.001	0.006	−0.298	−0.001–0.012	0.082
Age^2^	−0.00007	−0.061	−0.0002–0.000009	0.083					−0.00009	−0.072	−0.0002–−0.00003	0.005
Screen size	0.004	0.084	−0.001–0.008	0.098	0.002	0.063	−0.0001–0.005	0.062				
Delay					−0.002	−0.192	−0.002–−0.002	<0.001	−0.004	−0.223	−0.007–−0.002	<0.001
Delay^2^									0.00003	0.037	0.000004–0.00005	0.023
*Random effects*								
σ^2^	0.012					.012					0.015				
τ_00_	0.014 _UserID_					0.009 _UserID_					0.010 _UserID_				
τ_11_	0.000003 _UserID.days_since_baseline_										0.000002 _UserID.days_since_baseline_				
ρ_01_	0.054 _UserID_										0.220 _UserID_				
ICC	0.639					0.428					0.514				
N	212 _UserID_					395 _UserID_					237 _UserID_				
Observations	1,325					2,625					1,660				
Marginal/Conditional R^2^	0.134 / 0.687					0.167 / 0.523					0.107 / 0.565				

*The procedure leading to these final models is described in the Statistical Analyses, and all calculated models are included in Supplementary Material*.

### Important Factors for Unsupervised Assessments

One key difference between in-clinic assessments and remote digital cognitive assessments is the lack of a standardized testing environment in the latter scenario. Consequently, in order to interpret measures derived from remote digital cognitive assessments, everyday factors need to be recorded and their influence needs to be investigated, and controlled if necessary. Here we focused on (i) the time of day during the cognitive test, (ii) the time between encoding and retrieval for the delayed memory tasks and the (iii) screen size of the mobile device.

First, 73.6% of individual test sessions were performed between 8 a.m. and 8 p.m. (mean 2.38 PM, SD = 5 h 2 min). Using linear-mixed modeling analysis, we did not observe a significant influence of the time of day on cognitive performance in this study (see Supplementary Material). Second, participants were asked and reminded to complete the second phase of each test after its release following a 24-h delay. The resulting mean delay between the phases was 40.2 h, with hardly any difference between tasks, i.e., between encoding and retrieval in the ORR and CSR tasks as well as the time between the first and second half of the MDT-OS (see Table 1 for task-specific delays). Linear-mixed models revealed a significant influence of the delay between phases on memory performance in all three tasks (see Figure 4 and Table 2).

Mobile devices used in this study had a screen diagonal between 9 and 32.9 cm (mean = 14 cm, SD = 4.4 cm) indicating the use of smartphones as well as tablet computers. Linear-mixed models hinted toward better memory performance in MDT-OS and ORR with bigger screen size (see Table 2).

Additionally, we recorded perceptual discrimination, concentration, distraction and subjective memory performance. Perceptual discrimination performance showed a mean of 90.5%. Across all three cognitive tasks, participants reported high concentration levels during the task (see Table 1; mean phase 1 = 4, mean phase 2 = 3.6, which translates to good concentration), and middling to high subjectively rated task performance (mean phase 1 = 4, mean phase 2 = 2.9). While mean concentration levels were similar across tasks and phases, subjective task performance ratings indicated higher subjective task difficulty for the MDT-OS in general, and for the delayed retrieval of ORR and CSR. No distractions were reported during 75% of the completed sessions across individuals, however, most distractions occurred during MDT-OS, and the fewest distractions occurred during the retrieval phases of ORR and CSR, possibly because they are considerably shorter than the other sessions. See Table 1 for detailed stratification of all these measures.

### Attrition and Moderating Factors

**Figure 5:**
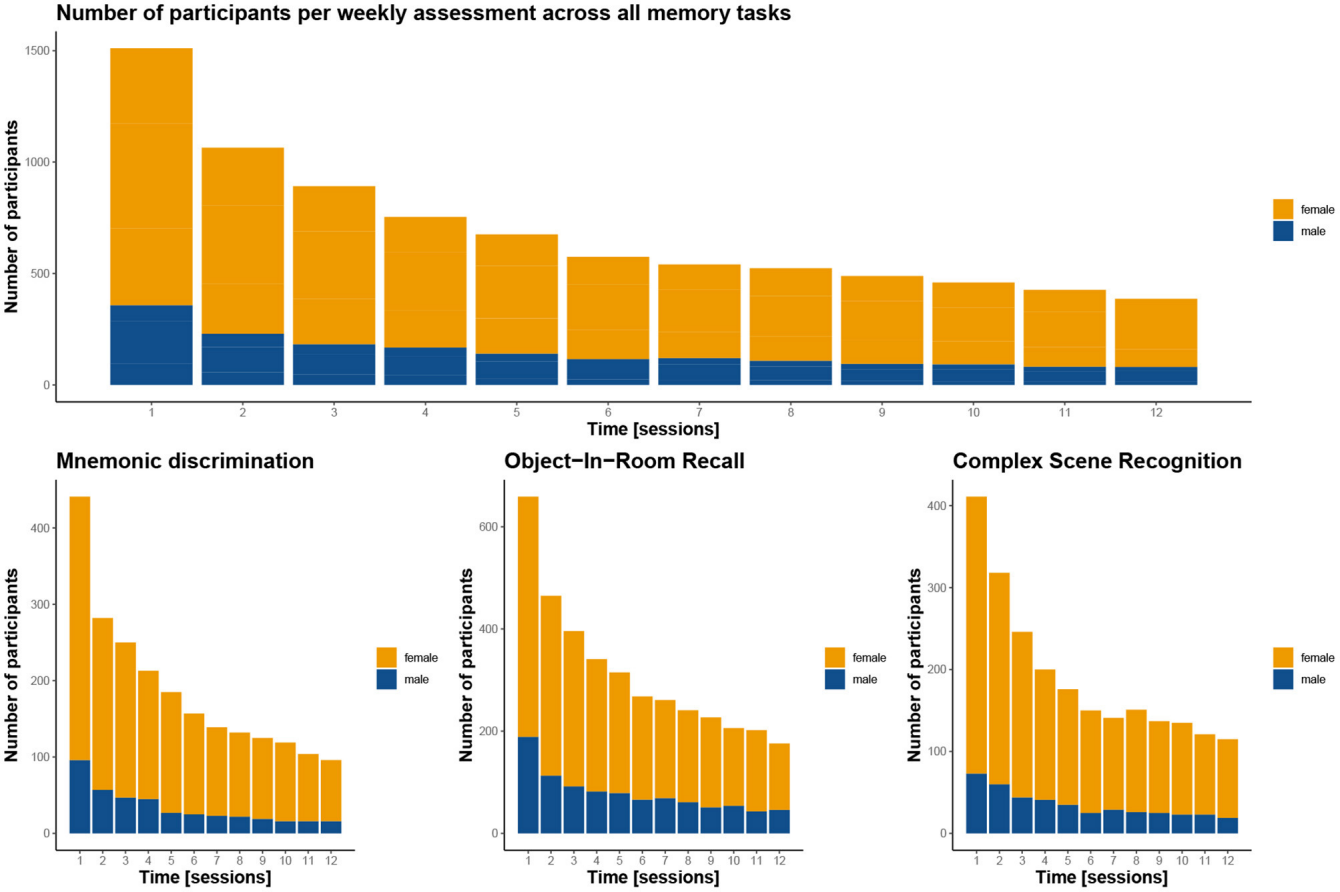
Participant completion rates by test session. The graphs demonstrate for how many sessions the participants contributed to the study overall (top panel) and for the three memory tasks (bottom panel). Notably, in all three tasks, there is a gross initial loss after the first session, but dropout is greatly reduced in the weeks after. Women are depicted in orange, men in blue. Note, that the top panel includes 138 participants twice as they contributed to two of the tasks, resulting in a total of 1,545 instead of the 1,407 participants enrolled in the study.

The average time participants stayed in the study was 44 days, and varied only slightly between the three memory tasks (see Table 1). Following an immediate loss of about half the participants after the first test session, the drop-out rate was significantly reduced in the following weeks for all three memory tasks, which can be easily assessed in Figure 5. More importantly, individuals contributed data to 4 complete sessions on average, with greater engagement in ORR and CSR (~5 sessions), and less engagement in MDT-OS (~4 sessions; see Table 1). Given that participants were supposed to take a weekly test, we analyzed compliance with the protocol by calculating the percentage of tests taken within their total time in the study, i.e., if participants completed their last session in week 10, they should have completed 10 sessions in total to receive a 100% score for compliance with protocol. Overall, participants complied with the protocol 77.5%, with hardly any differences between tasks (see Table 1).

**Figure 6:**
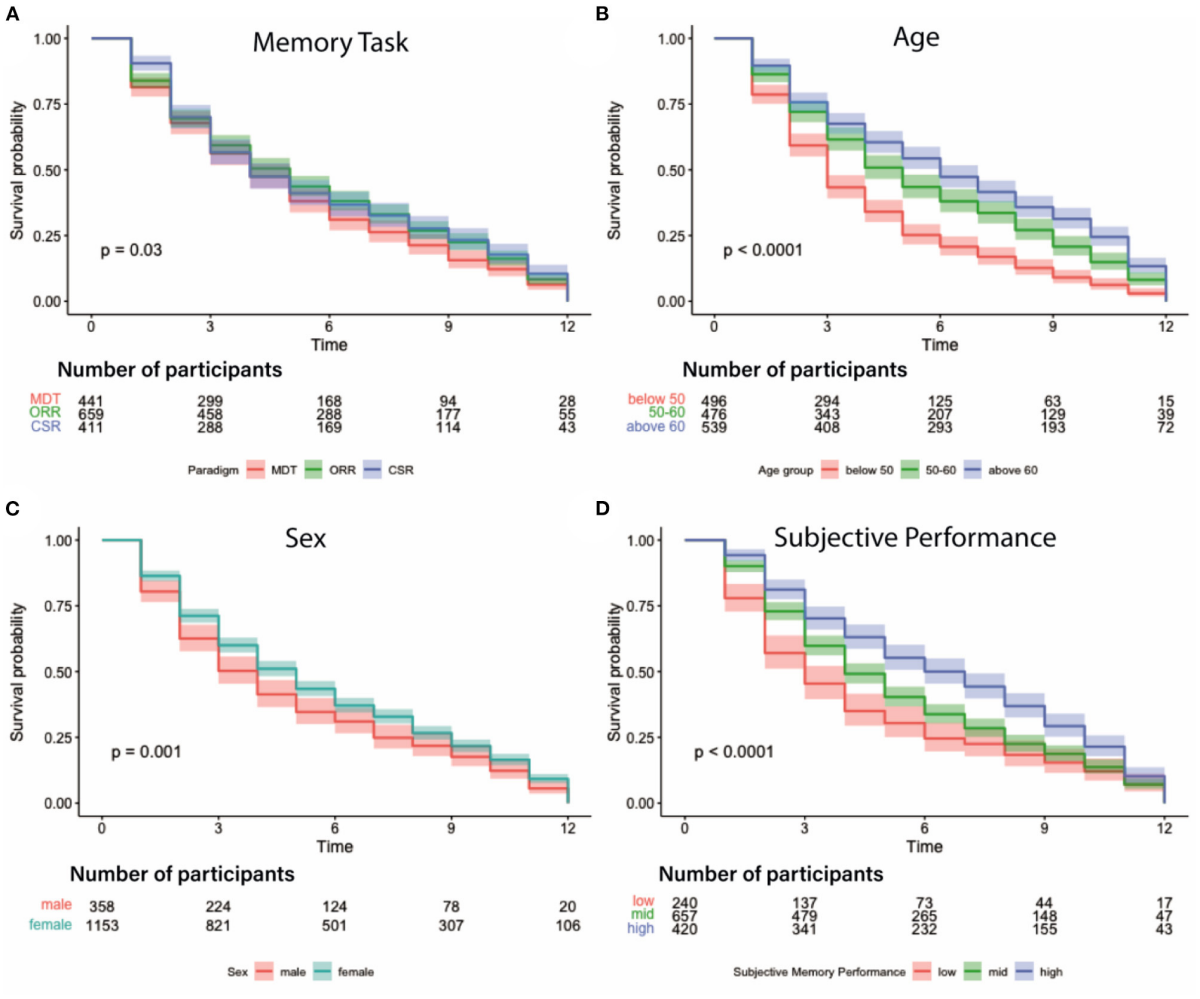
Participation survival analysis. Graphs depict different factors influencing participant dropout over the course of 12 weeks. **(A)** Attrition depending on memory task suggests that participants of the CSR remained in the study longer than in the other tasks; MDT-OS in red, ORR in green, CSR in blue. **(B)** Attrition per age group with participants older than 50 contributing longer than those below 50; groups are split according to 33/66% quantiles: below 50 in red, 50–60 in green, above 60 in blue. **(C)** Attrition by sex indicating that women stayed in the study for longer; male in red, female in blue. **(D)** Attrition based on subjective task performance with participants engaging longer the better they judged their performance on a 5-point scale from 1:very bad to 5:very good; groups were formed using these cut-offs: low - ratings up to 2 in red, mid - ratings between 2 and 3 in green, high - ratings above 3 in blue.

Survival analysis of the number of contributed sessions revealed several factors significantly associated with participant retention. Note, that here contributed sessions do not necessarily have to be complete, and numbers are therefore slightly higher than the average 4 complete sessions. As already conceivable from the mean retention time and contributed sessions, we observed a significant effect of memory task which suggests that individuals using the MDT-OS left the study sooner than participants of the ORR and the CSR (*p* < 0.03, see Figure 6A). Furthermore, we found a significant effect of age (18–49 years: mean = 4.1 sessions, 50–60 years: mean = 5.6 sessions, above 60 years: mean = 6.4 sessions; *p* < 0.0001) and sex (males: mean = 4.8 sessions, females: mean = 5.6 sessions; *p* = 0.001) suggesting that females and older individuals showed higher retention (see Figures 6B,C). We also observed that individuals engaged in more sessions, the better their subjective memory performance was (low: mean=4.5 sessions, mid: mean = 5.4 sessions, high: mean = 6.6 sessions; *p* < 0.0001, see Figure 6D). Objective memory performance, however, was no significant predictor (low: mean=5.3 sessions, mid: mean = 5.5 sessions, high: mean = 5.5 sessions; *p* = 0.88).

### Practice Effects and Retest Reliability

Repeated memory testing commonly results in practice effects, because of task familiarity, and because the same stimuli are used repeatedly (15–17). Here we tried to minimize practice effects by utilizing independent parallel stimulus sets where no stimulus was repeated across sessions. However, practice effects can also arise from procedural practice such as getting familiar with the task structure or developing task strategies. Using linear-mixed modeling, we only observed moderate practice effects in the MDT-OS, and ORR with a linear increase in performance with repeated sessions (see Table 2), and no effects for CSR.

Finally, we were interested in the retest reliability of the different memory tasks. Pearson correlation coefficients showed retest reliability of a single test session (session 2 vs. session 3) of 0.74, 0.64 and 0.68 for MDT-OS, ORR and CSR respectively. To account for daily form, we also used an average across two sessions (mean of session 1 and 2 vs. mean of session 3 and 4), and found good retest reliability, which was highest for MDT-OS (0.81) followed by CSR (0.79) and ORR (0.72).

## Discussion

In this large-scale study, we set out to assess the feasibility of remote and unsupervised digital memory assessments via mobile devices such as smartphones and tablets. We utilized three novel memory paradigms including delayed recall phases for weekly digital memory assessments in a *Citizen Science* project with 1,407 individuals across Germany. First, while participants remained in the study for an average of 4 complete test sessions corresponding to 8 app interactions (phases) across a mean retention time of 6 weeks, factors such as older age, female sex and better subjective memory performance were identified as predictors for longer participant retention. Second, we found only minimal practice effects across weekly repeated tests in two of the three tasks likely due to the utilization of parallel stimulus sets, and we observed acceptable to good retest reliability across all memory tests. Third, while higher age was substantially associated with lower memory performance across all tasks, individuals with perceived subjective memory decline only presented objective lower mnemonic discrimination accuracy, but no impairment in object-in-room recall. Finally, we identified important factors that need to be incorporated in future studies utilizing unsupervised and remote memory assessments such as the time between encoding and retrieval. Taken together, our results demonstrate that remote digital assessments can be applied in an unsupervised fashion and seem suitable for longitudinal cognitive assessments in the future.

### Participation and Retention

Unsupervised digital cognitive assessments have the potential to transform the way scientists conduct biomedical research and might enable partly digital and decentralized clinical trials where cognition represents a critical measure. Here, a total of 1,407 individuals participated in our three independent studies and while all three studies resulted in similar demographics and individuals across the almost entire adult age range, all had in common that more than 70% of participants were female (75.6% across studies). This is in line with a similar gender bias in earlier digital Citizen Science studies focusing on health (18), and other areas (19, 20) suggesting that specific strategies need to be taken to reach a more diverse participant sample in future studies.

Remote digital assessments have been hampered by substantial participant attrition in earlier remote studies. A recent meta-analysis pooling data across eight digital health studies reported median participant retention of 5.5 days with 2 active interactions within a time of 12 weeks, but with high variations across studies of 2–26 days (5). Here, we found mean retention of 44 days, or four sessions, that is, at least 8 successfully completed active app interactions. Following severe and immediate drop out in the first assessments, retention became more stable after participants had contributed a couple of sessions. We could identify several significant influences by exploring factors that were associated with higher retention times using survival analyses. They revealed that female participants stayed in the study longer than male participants. Similarly, individuals above age 50 showed higher retention times compared to those younger than 50, a result that is in direct agreement with the recent meta-analysis (5). Previous findings from longitudinal in-clinic memory studies suggest that those with higher levels of cognitive performance returned for follow up assessments more frequently than individuals with lower cognitive performance (21). Similarly, participants in a remote study performed more sessions, the better their performance was (22). While actual memory performance was no predictor of retention in our study, better subjective memory performance was clearly associated with higher retention times. Interestingly, subjective task performance in the MDT-OS was rated lower and retention was shorter compared to ORR and CSR. This might suggest that specific feedback strategies to increase subjective task performance levels might help to increase long term retention (23). Of note, Pratap and colleagues report that retention times are in general higher in studies with personal contact between participants and study personnel. While there was no personal contact in our study, we would similarly expect higher retention times in research studies with personal contact and additional on-site examinations.

### High Usability of Remote and Unsupervised Assessments

Regarding the usability of remote cognitive assessments, we observed that 75% of test sessions across all individuals and tasks were completed without any distraction. However, we do not yet know what qualified as distraction and what type of distractions were happening, which has to be investigated in future studies. Apparently, however, participants succeeded to complete tasks in a quiet environment as the clear majority of cases reported high concentration rates during task performance. Tasks were completed from mobile devices with a wide range of screen sizes showing that beside smartphones also tablet computers were used in the study. Participants did also comply with the protocol as observed in the low percentage of missed sessions, and a moderate extension of the delay between the first and the second phase of each test session. In addition, while there were some differences between studies and tasks, perceived task difficulty was neither too hard nor too easy.

### Minimal Practice Effects and Good Retest Reliability

Most memory tests come with a very limited set of parallel test versions which hamper repeated assessment of memory performance over time (24–26). Carefully optimized alternate test versions with well-matched stimulus material can attenuate or even eradicate practice effects associated with repeated testing (1, 15, 16, 27).

Repeated tests on a weekly basis for up to 12 weeks represent a significant challenge when it comes to associated practice effects. However, our results indicate only minimal or no practice effects in the present tasks due to independent alternate test versions where not one stimulus was presented twice during the study. While there were no practice effects observed for CSR, there were only minimal practice gains in the MDT-OS and ORR. These differences might result from the differential improvements that can be gained from a specific strategy. Over time, and in particular across the first test sessions, individuals might improve their strategy by slowly getting aware of the most likely changes to appear. This might partly underly the practice effect and can likely be circumvented by adding several initial tests at baseline.

We also assessed both retest reliability of one single test result as well as retest reliability derived from averaging across two successive test sessions. While individual estimates were ranging between 0.64 and 0.74, representing moderate reliability, averaging across only two short test sessions resulted already in good retest reliability (0.72–0.81). This is well in the range of established memory assessment batteries such as ADAS-cog delayed recall (28, 29) (0.76–0.78) or the California Verbal Learning task (CVLT-II, 0.61–0.73) (30) with the notable difference that those have been acquired in supervised in-clinic assessments in a standardized testing environment. The observed retest reliability supports application of longitudinal digital assessments in future studies.

### Worse Task Performance With Higher Age and Subjective Memory Decline

Episodic memory performance has been reliably shown to be lower in older age groups (31–33). Using unsupervised remote digital assessments in a Citizen Science sample we indeed observed significant differences in memory performance across all three paradigms in higher age groups consistent with earlier work using the same paradigms in supervised (6, 8) and unsupervised settings (34). Age is also associated with various factors that might be causal for memory impairment such as cerebrovascular lesions or Alzheimer's disease pathology (35). Given the remote and unsupervised setting of our Citizen Science project we cannot rule out potential effects of other associated but uncontrolled variables and interpret the age-related memory impairment thus in the light of a proxy for age-related health factors.

Subjective cognitive and memory decline has been suggested as an early symptom of Alzheimer's disease (36). In two sub-studies of our Citizen Science cohort, we asked individuals whether they have experienced cognitive decline in the 5 years before entering the study (14). Based on their response, individuals where subdivided into two groups, those with subjectively stable, and those with subjectively declining memory. Our results indicate that while there was no difference between stable and declining groups in the ORR, those with subjective memory decline showed overall reduced performance in the MDT-OS. However, while this effect could be detected in overall performance levels, there was no significant interaction with time suggesting that both groups did not show differences in longitudinal decline over 12 weeks. Future longitudinal studies across even longer time intervals are needed in well characterized aging or dementia research cohorts to investigate whether mobile memory assessments are also suitable to detect longitudinal decline. Taken together, while age-related memory impairment as well as differences between subjectively stable and declining groups are as expected, future studies combining in-clinic and unsupervised remote assessments need to define convergent and discriminant validity in detail in comparison with established neuropsychological assessment batteries. Recently, we could show that the same memory tests used here, combined to a composite score, are suitable to detect mild cognitive impairment in a memory-clinic population, and show high construct validity with an in-clinic neuropsychological assessment (37).

### Influence of Time to Retrieval and Device Screen Size but Not Time of Day

Several factors in our everyday life are expected to influence our cognitive and memory performance. Memory performance varies throughout the day (38, 39), throughout seasons (40) but has also been reported to depend on sleep duration (41) and daily stress levels (42). Participants completed our tasks around the clock but by far the most tests were completed during the day. While we did not observe a significant influence of the time of day on memory performance, other researchers have (43). Thus, future studies might want to recommend or restrict task completion to a specific time range, and should further investigate this relationship. Additionally, we investigated effects of sleep duration and cold symptoms on memory performance, but did not observe any. Potentially, our self-report scales may need to be extended, for example with sleep quality, given that earlier reports in the literature have found sleep impacts cognitive performance (41). Alternatively, actigraphy data may be used to reduce the active effort participants have to put in the completion of the remote assessments.

In long term memory assessment with a delay period, the length of the delay between encoding and retrieval also affects memory performance (44). In accordance with these earlier reports, we found that time between encoding and retrieval in both delayed memory tasks (ORR and CSR) was negatively related to memory performance where longer delay times were associated with lower memory performance. However, both results come with limitations due to the non-interventional nature of our study. While studies actively manipulating delay intervals can assign their findings almost purely to the variation in delay times, we must also consider the possibility that those individuals who postponed their task to later times had worse memory function in general.

In addition, technical factors associated with different mobile devices have the potential to influence measures of memory performance and have thus to be taken into account in unsupervised remote digital assessments. Earlier reports have stressed the importance of differences between mobile devices when it comes to measuring reaction times (4, 45), however, different screen sizes may also affect accuracy measures of memory performance in visual memory tasks. Here, we found evidence for improved memory performance with bigger screens in two of the three tasks. Taken together, for many of the investigated factors in our study, such as sleep, cold symptoms, and time of day, we did not observe indications for substantial confounding of task performance, although they have shown to do so in other tasks in the literature. While this fact needs to be treated with caution, and preemptive measures may be taken anyhow (e.g., restricting time of day, gathering actigraphy data when available), it is promising, given that remote digital assessments cannot guarantee the controlled environment of in-clinic testing. However, the delay between encoding and retrieval was an important factor that clearly influenced task performance, and should best be kept constant and short. Therefore, we recommend additional reminders and showing participants the exact times of future tests from the beginning as well as actual restrictions to the time of retrieval in remote digital assessments.

### Limitations

Beside the strengths of our study there are also several limitations. First, while our unsupervised and remote study design allowed us to investigate test reliability and validity regarding age effects and subjective memory decline, these are not comparable with traditional standardized neuropsychological in-clinic assessments. Thus, more studies are needed to define validity with established neuropsychological test batteries. Second, due to a missing questionnaire on years of education, we are not able to investigate how much of the effects and their variability is due to different levels of education and socioeconomic status. Third, while we could identify factors that were associated with retention times, this study did not allow us to further investigate what led individuals to leave the study before the end. It will be critical to understand study drop-outs in more detail in the future in controlled study settings and for example investigate how individuals perceived the study schedule as well as how they reacted to reminders via push notifications. This might allow to further decrease study drop-outs and increase long-term engagement. Fourth, we decided to pseudo-randomize the study sample into three different sub studies completing different memory tasks in order to maximize the number of consecutive test session to have the necessary statistical power for our longitudinal analyses. However, on the contrary that prevents us from direct comparisons between memory tasks and the possibility to explore the combination of test scores between different tasks in composite measures. The few overlapping participants were not enough to assess these comparisons. Finally, although our randomization strategy led to roughly comparable subsamples with respect to age and gender balance, it resulted in an overall bias toward more females and is, thus, not representative of the general population.

## Conclusion

Taken together, our results show that repeated unsupervised digital assessments are feasible and allow the collection of cognitive data within the population. We could identify important factors that were associated with memory performance or digital measures thereof that need to be incorporated in future studies using digital remote memory assessments.

## Data Availability Statement

The datasets presented in this article are not readily available because anonymized data will be shared by request from academic investigators for the purpose of replicating results presented in this article as long as data transfer is in agreement with EU legislation on the general data protection regulation and decisions by the Ethical Review Board of the Medical Faculty at Otto-von-Guericke University Magdeburg, which should be regulated in a material transfer agreement. Requests to access the datasets should be directed to david.berron@dzne.de.

## Ethics Statement

The study was approved by the Ethics Committee of the Medical Faculty of the Otto-von-Guericke-University Magdeburg. The patients/participants provided their written informed consent to participate in this study.

## Author Contributions

DB, OB, MH, AS, ST, FJ, MW, and ED contributed to conception and design of the study. DB organized the database and wrote the first draft of the manuscript. DB, GZ, and PV performed the statistical analysis. PV and GZ wrote sections of the manuscript. All authors contributed to manuscript revision, read, and approved the submitted version.

## Conflict of Interest

DB and ED are co-founders and hold shares of neotiv GmbH, OB is a full-time employee of neotiv GmbH. GZ has been a part time employee at neotiv GmbH from 05/2019 to 10/2019. The remaining authors declare that the research was conducted in the absence of any commercial or financial relationships that could be construed as a potential conflict of interest.

## Publisher's Note

All claims expressed in this article are solely those of the authors and do not necessarily represent those of their affiliated organizations, or those of the publisher, the editors and the reviewers. Any product that may be evaluated in this article, or claim that may be made by its manufacturer, is not guaranteed or endorsed by the publisher.
