# Supplementary material for: Feasibility of Digital Memory Assessments in an Unsupervised and Remote Study Setting
Source: Front Digit Health. 2022 May 26;4:892997. doi: 10.3389/fdgth.2022.892997 (PMC9199443; doi:10.3389/fdgth.2022.892997)
Supplement: Supplementary file 1 [file Table_1.pdf]

FEASIBILITY OF DIGITAL MEMORY ASSESSMENTS IN AN UNSUPERVISED AND REMOTE STUDY SETTING

Supplementary material

David Berron[1,3,10], Gabriel Ziegler[1,2,10], Ornella Billette[1,2,10], Jeremie Güsten[1,2], Xenia Grande[1,2], Paula Vieweg[1], Michael T. Heneka[4,5], Anja Schneider[4,5], Stefan Teipel[6,7], Frank Jessen[4,8], Michael Wagner[4,5], Emrah Düzel[1,2,9,10]

[1] German Center for Neurodegenerative Diseases (DZNE), Magdeburg, Germany,

[2] Institute of Cognitive Neurology and Dementia Research (IKND), Magdeburg, Germany

[3] Clinical Memory Research Unit, Department of Clinical Sciences Malmö, Lund University, Lund, Sweden

[4] German Center for Neurodegenerative Diseases (DZNE), Bonn, Germany

[5] Department of Neurodegeneration and Geriatric Psychiatry, University of Bonn, Bonn, Germany

[6] Department of Psychosomatic Medicine, Rostock University Medical Center, Rostock, Germany

[7] German Center for Neurodegenerative Diseases, Rostock, Germany

[8] Department of Psychiatry, University Hospital Cologne, Cologne, Germany

[9] University College London, Institute of Cognitive Neuroscience, London, United Kingdom

[10] neotiv GmbH, Magdeburg, Germany

Object-in-Room Recall

	linear effects of interest			+ quadratic effects			- non-significant effects + interactions			- non-significant effects			FINAL: linear effects of interest - non-significant effects (AIC based)			+ slope			+ cold symptoms			+ sleep			+ subjective memory decline		
Predictors	Estimates	CI	p	Estimates	CI	p	Estimates	CI	p	Estimates	CI	p	Estimates	CI	p	Estimates	CI	p	Estimates	CI	p	Estimates	CI	p	Estimates	CI	p
(intercept)	0.86082	0.79618 – 0.92547	**<0.001**	0.91394	0.75616 – 1.07171	**<0.001**	0.96370	0.75262 – 1.17478	**<0.001**	0.91490	0.84900 – 0.98080	**<0.001**	0.86371	0.80701 – 0.92040	**<0.001**	0.86500	0.80840 – 0.92160	**<0.001**	0.86618	0.79637 – 0.93599	**<0.001**	0.82391	0.73002 – 0.91781	**<0.001**	0.84994	0.79120 – 0.90867	**<0.001**
time	0.00027	0.00010 – 0.00043	**0.002**	0.00058	0.00003 – 0.00113	**0.040**	0.00039	-0.00051 – 0.00130	0.394	0.00028	0.00011 – 0.00044	**0.001**	0.00027	0.00010 – 0.00044	**0.002**	0.00026	0.00008 – 0.00044	**0.004**	0.00025	0.00002 – 0.00047	**0.032**	0.00027	0.00010 – 0.00044	**0.002**	0.00021	0.00004 – 0.00039	**0.017**
age	-0.00472	-0.00560 – -0.00384	**<0.001**	-0.00620	-0.01174 – -0.00065	**0.028**	-0.00589	-0.00929 – -0.00249	**0.001**	-0.00510	-0.00606 – -0.00414	**<0.001**	-0.00475	-0.00563 – -0.00388	**<0.001**	-0.00475	-0.00562 – -0.00388	**<0.001**	-0.00442	-0.00549 – -0.00334	**<0.001**	-0.00479	-0.00566 – -0.00392	**<0.001**	-0.00468	-0.00559 – -0.00377	**<0.001**
sex	0.01301	-0.01172 – 0.03775	0.302	0.01360	-0.01128 – 0.03847	0.284																					
delay	-0.00180	-0.00209 – -0.00152	**<0.001**	-0.00361	-0.00502 – -0.00221	**<0.001**	-0.00331	-0.00471 – -0.00191	**<0.001**	-0.00332	-0.00472 – -0.00192	**<0.001**	-0.00178	-0.00206 – -0.00150	**<0.001**	-0.00177	-0.00205 – -0.00149	**<0.001**	-0.00175	-0.00211 – -0.00140	**<0.001**	-0.00178	-0.00206 – -0.00150	**<0.001**	-0.00179	-0.00209 – -0.00150	**<0.001**
time of day	-0.00065	-0.00159 – 0.00029	0.172	0.00267	-0.00248 – 0.00781	0.310																					
screen size	0.00230	-0.00002 – 0.00461	0.052	0.00234	0.00003 – 0.00464	**0.047**	-0.00170	-0.01695 – 0.01354	0.827	0.00224	-0.00006 – 0.00454	0.057	0.00220	-0.00011 – 0.00451	0.062	0.00207	-0.00024 – 0.00438	0.079	0.00124	-0.00159 – 0.00406	0.390	0.00218	-0.00012 – 0.00449	0.063	0.00354	0.00106 – 0.00602	**0.005**
time^2				-0.00000	-0.00001 – 0.00000	0.249																					
age^2				0.00001	-0.00004 – 0.00006	0.596																					
delay^2				0.00002	0.00000 – 0.00003	**0.011**	0.00000	-0.00002 – 0.00002	0.784	0.00000	-0.00002 – 0.00002	0.769															
time of day^2				-0.00013	-0.00032 – 0.00006	0.183																					
time * age							-0.00000	-0.00002 – 0.00001	0.795																		
age * delay^2							0.00000	-0.00000 – 0.00000	0.088	0.00000	-0.00000 – 0.00000	0.087															
age * screen size							0.00006	-0.00018 – 0.00031	0.608																		
cold symptoms																			-0.01371	-0.04175 – 0.01434	0.338						
sleep																						0.00599	-0.00528 – 0.01726	0.297			
subjective memory decline																									-0.01416	-0.03939 – 0.01107	0.271
Random Effects																											
σ^2	0.01174			0.01172			0.01173			0.01172			0.01175			0.01151			0.01233			0.01175			0.01181		
τ_{00}	0.00880 $_{UserID}$			0.00873 $_{UserID}$			0.00875 $_{UserID}$			0.00872 $_{UserID}$			0.00879 $_{UserID}$			0.00883 $_{UserID}$			0.00902 $_{UserID}$			0.00879 $_{UserID}$			0.00865 $_{UserID}$		
τ_{11}																0.00000 $_{UserID.days\_since\_baseline}$											
ρ_{01}																-0.12043 $_{UserID}$											
ICC	0.42835			0.42697			0.42717			0.42651			0.42803			0.44003			0.42242			0.42787			0.42282		
N	395 $_{UserID}$			395 $_{UserID}$			395 $_{UserID}$			395 $_{UserID}$			395 $_{UserID}$			395 $_{UserID}$			276 $_{UserID}$			395 $_{UserID}$			350 $_{UserID}$		
Observations	2625			2625			2625			2625			2625			2625			1688			2625			2396		
Marginal R^2 / Conditional R^2	0.168 / 0.524			0.170 / 0.524			0.170 / 0.524			0.170 / 0.524			0.167 / 0.523			0.166 / 0.533			0.148 / 0.508			0.168 / 0.524			0.168 / 0.520		
AIC	-3445.722			-3366.285			-3378.447			-3420.006			-3467.185			-3467.220			-2102.539			-3457.791			-3151.779		

Mnemonic Discrimination Task of Objects and Scenes

	linear effects of interest			+ quadratic effects			- non-significant effects + interactions			- non-significant effects		
Predictors	Estimates	CI	p	Estimates	CI	p	Estimates	CI	p	Estimates	CI	p
(intercept)	0.80870	0.69957 – 0.91782	**<0.001**	0.57392	0.30895 – 0.83889	**<0.001**	0.60605	0.33133 – 0.88077	**<0.001**	0.57898	0.32296 – 0.83499	**<0.001**
time	0.00051	0.00025 – 0.00078	**<0.001**	0.00035	-0.00048 – 0.00118	0.414	0.00083	0.00011 – 0.00155	**0.024**	0.00046	0.00020 – 0.00072	**0.001**
age	-0.00563	-0.00712 – -0.00415	**<0.001**	0.00341	-0.00627 – 0.01310	0.489	0.00331	-0.00690 – 0.01352	0.525	0.00280	-0.00669 – 0.01229	0.563
sex	-0.00554	-0.05673 – 0.04564	0.832	-0.01477	-0.06674 – 0.03720	0.577						
delay	-0.00032	-0.00071 – 0.00007	0.106	0.00104	-0.00096 – 0.00303	0.310						
time of day	0.00002	-0.00139 – 0.00143	0.978	-0.00259	-0.01029 – 0.00511	0.510						
screen size	0.00399	-0.00070 – 0.00868	0.096	0.00425	-0.00044 – 0.00893	0.076	0.00032	-0.01489 – 0.01554	0.967	0.00414	-0.00051 – 0.00880	0.081
time^2				0.00000	-0.00001 – 0.00001	0.710						
age^2				-0.00008	-0.00017 – 0.00000	0.064	-0.00009	-0.00021 – 0.00003	0.131	-0.00008	-0.00017 – 0.00001	0.078
delay^2				-0.00001	-0.00003 – 0.00001	0.175						
time of day^2				0.00010	-0.00018 – 0.00038	0.490						
time * age^2							-0.00000	-0.00000 – 0.00000	0.276			
age^2 * screen size							0.00000	-0.00000 – 0.00000	0.604			
cold symptoms												
sleep												
subjective memory decline												
Random Effects												
σ^2	0.01361			0.01361			0.01362			0.01362		
τ_{00}	0.01796 $_{UserID}$			0.01778 $_{UserID}$			0.01771 $_{UserID}$			0.01765 $_{UserID}$		
τ_{11}												
ρ_{01}												
ICC	0.56896			0.56647			0.56533			0.56453		
N	212 $_{UserID}$			212 $_{UserID}$			212 $_{UserID}$			212 $_{UserID}$		
Observations	1325			1325			1325			1325		
Marginal R^2 / Conditional R^2	0.135 / 0.627			0.151 / 0.632			0.151 / 0.631			0.149 / 0.629		
AIC	-1388.723			-1308.714			-1350.671			-1408.236		

	FINAL: + slope (AIC based)			+ cold symptoms			+ sleep			+ subjective memory decline		
Predictors	Estimates	CI	p	Estimates	CI	p	Estimates	CI	p	Estimates	CI	p
(intercept)	0.58714	0.34797 – 0.82632	**<0.001**	0.74737	0.45756 – 1.03718	**<0.001**	0.55287	0.25535 – 0.85039	**<0.001**	0.59473	0.34462 – 0.84484	**<0.001**
time	0.00042	0.00005 – 0.00079	**0.027**	0.00040	-0.00008 – 0.00088	0.101	0.00042	0.00005 – 0.00079	**0.027**	0.00045	0.00006 – 0.00085	**0.023**
age	0.00243	-0.00645 – 0.01131	0.592	-0.00292	-0.01401 – 0.00817	0.605	0.00273	-0.00630 – 0.01175	0.554	0.00256	-0.00670 – 0.01182	0.588
sex												
delay												
time of day												
screen size	0.00372	-0.00068 – 0.00812	0.098	0.00300	-0.00267 – 0.00867	0.300	0.00370	-0.00071 – 0.00811	0.100	0.00362	-0.00114 – 0.00838	0.136
time^2												
age^2	-0.00007	-0.00015 – 0.00001	0.083	-0.00003	-0.00013 – 0.00008	0.627	-0.00008	-0.00016 – 0.00001	0.076	-0.00007	-0.00016 – 0.00001	0.087
delay^2												
time of day^2												
time * age^2												
age^2 * screen size												
cold symptoms				-0.03185	-0.09243 – 0.02872	0.302						
sleep							0.00389	-0.01619 – 0.02397	0.704			
subjective memory decline										-0.04399	-0.08777 – -0.00022	**0.049**
Random Effects												
σ^2	0.01159			0.01129			0.01159			0.01173		
τ_{00}	0.01386 $_{UserID}$			0.01384 $_{UserID}$			0.01396 $_{UserID}$			0.01422 $_{UserID}$		
τ_{11}	0.00000 $_{UserID.days\_since\_baseline}$			0.00000 $_{UserID.days\_since\_baseline}$			0.00000 $_{UserID.days\_since\_baseline}$			0.00000 $_{UserID.days\_since\_baseline}$		
ρ_{01}	0.05351 $_{UserID}$			-0.00410 $_{UserID}$			0.04979 $_{UserID}$			0.03795 $_{UserID}$		
ICC	0.63910			0.65249			0.63952			0.63998		
N	212 $_{UserID}$			135 $_{UserID}$			212 $_{UserID}$			191 $_{UserID}$		
Observations	1325			903			1325			1231		
Marginal R^2 / Conditional R^2	0.134 / 0.687			0.144 / 0.703			0.134 / 0.688			0.150 / 0.694		
AIC	-1482.894			-1002.448			-1473.709			-1357.915		

Complex Scene Recognition

	linear effects of interest			+ quadratic effects			- non-significant effects + interactions			- non-significant effects			FINAL: + slope (AIC based)			+ cold symptoms			+ sleep		
Predictors	Estimates	CI	p	Estimates	CI	p	Estimates	CI	p	Estimates	CI	p	Estimates	CI	p	Estimates	CI	p	Estimates	CI	p
(intercept)	0.66897	0.56650 – 0.77144	**<0.001**	0.52078	0.32782 – 0.71373	**<0.001**	0.53914	0.35538 – 0.72289	**<0.001**	0.53884	0.35558 – 0.72209	**<0.001**	0.53641	0.36622 – 0.70660	**<0.001**	0.59166	0.35970 – 0.82362	**<0.001**	0.50923	0.26366 – 0.75479	**<0.001**
time	-0.00021	-0.00045 – 0.00003	0.085	-0.00006	-0.00090 – 0.00078	0.887	-0.00007	-0.00080 – 0.00066	0.851	-0.00021	-0.00045 – 0.00003	0.087	-0.00028	-0.00059 – 0.00004	0.083	-0.00032	-0.00073 – 0.00010	0.135	-0.00028	-0.00059 – 0.00004	0.083
age	-0.00354	-0.00479 – -0.00229	**<0.001**	0.00422	-0.00268 – 0.01111	0.231	0.00546	-0.00150 – 0.01241	0.124	0.00557	-0.00135 – 0.01250	0.115	0.00572	-0.00073 – 0.01217	0.082	0.00391	-0.00517 – 0.01299	0.398	0.00602	-0.00074 – 0.01278	0.081
sex	0.02524	-0.01854 – 0.06902	0.258	0.02229	-0.02112 – 0.06569	0.314															
delay	-0.00197	-0.00241 – -0.00153	**<0.001**	-0.00466	-0.00691 – -0.00240	**<0.001**	-0.00426	-0.00648 – -0.00204	**<0.001**	-0.00426	-0.00648 – -0.00204	**<0.001**	-0.00440	-0.00658 – -0.00222	**<0.001**	-0.00378	-0.00668 – -0.00088	**0.011**	-0.00440	-0.00658 – -0.00222	**<0.001**
time of day	-0.00092	-0.00237 – 0.00053	0.211	0.00422	-0.00380 – 0.01224	0.302															
screen size	0.00277	-0.00127 – 0.00682	0.179	0.00227	-0.00176 – 0.00630	0.269															
time^2				-0.00000	-0.00001 – 0.00001	0.713															
age^2				-0.00008	-0.00014 – -0.00001	**0.024**	-0.00009	-0.00015 – -0.00002	**0.013**	-0.00009	-0.00015 – -0.00002	**0.010**	-0.00009	-0.00015 – -0.00003	**0.005**	-0.00008	-0.00017 – 0.00001	0.078	-0.00009	-0.00016 – -0.00003	**0.006**
delay^2				0.00003	0.00000 – 0.00005	**0.018**	0.00002	-0.00000 – 0.00005	0.071	0.00002	0.00000 – 0.00005	**0.035**	0.00003	0.00000 – 0.00005	**0.023**	0.00002	-0.00001 – 0.00005	0.166	0.00003	0.00000 – 0.00005	**0.023**
time of day^2				-0.00020	-0.00048 – 0.00009	0.182															
time * age^2							-0.00000	-0.00000 – 0.00000	0.692												
age^2 * delay^2							0.00000	-0.00000 – 0.00000	0.902												
cold symptoms																-0.01372	-0.09391 – 0.06646	0.737			
sleep																			0.00281	-0.01547 – 0.02109	0.763
Random Effects																					
σ^2	0.01659			0.01655			0.01669			0.01666			0.01537			0.01580			0.01537		
τ_{00}	0.01388 UserID			0.01350 UserID			0.01400 UserID			0.01402 UserID			0.01015 UserID			0.00954 UserID			0.01022 UserID		
τ_{11}													0.00000 UserID.days_since_baseline			0.00000 UserID.days_since_baseline			0.00000 UserID.days_since_baseline		
ρ_{01}													0.21956 UserID			0.23404 UserID			0.21926 UserID		
ICC	0.45552			0.44926			0.45611			0.45689			0.51354			0.50232			0.51466		
N	232 UserID			232 UserID			237 UserID			237 UserID			237 UserID			136 UserID			237 UserID		
Observations	1626			1626			1660			1660			1660			951			1660		
Marginal R^2 / Conditional R^2	0.101 / 0.510			0.121 / 0.516			0.111 / 0.517			0.111 / 0.517			0.107 / 0.565			0.121 / 0.563			0.107 / 0.567		
AIC	-1532.784			-1459.336			-1474.861			-1547.677			-1591.895			-845.156			-1582.470		
